# Design and Validation of a Human Brain Endothelial Microvessel-on-a-Chip Open Microfluidic Model Enabling Advanced Optical Imaging

**DOI:** 10.3389/fbioe.2020.573775

**Published:** 2020-09-28

**Authors:** Mootaz M. Salman, Graham Marsh, Ilja Kusters, Matthieu Delincé, Giuseppe Di Caprio, Srigokul Upadhyayula, Giovanni de Nola, Ronan Hunt, Kazuka G. Ohashi, Taylor Gray, Fumitaka Shimizu, Yasuteru Sano, Takashi Kanda, Birgit Obermeier, Tom Kirchhausen

**Affiliations:** ^1^Department of Cell Biology, Harvard Medical School, Boston, MA, United States; ^2^Program in Cellular and Molecular Medicine, Boston Children’s Hospital, Boston, MA, United States; ^3^Biogen, Cambridge, MA, United States; ^4^Yamaguchi University Graduate School of Medicine, Ube, Japan; ^5^Department of Pediatrics, Harvard Medical School, Boston, MA, United States

**Keywords:** blood-brain barrier (BBB), capillary, microvessel, shear stress, microfluidics, live cell imaging

## Abstract

We describe here the design and implementation of an *in vitro* microvascular open model system using human brain microvascular endothelial cells. The design has several advantages over other traditional closed microfluidic platforms: (1) it enables controlled unidirectional flow of media at physiological rates to support vascular function, (2) it allows for very small volumes which makes the device ideal for studies involving biotherapeutics, (3) it is amenable for multiple high resolution imaging modalities such as transmission electron microscopy (TEM), 3D live fluorescence imaging using traditional spinning disk confocal microscopy, and advanced lattice light sheet microscopy (LLSM). Importantly, we miniaturized the design, so it can fit within the physical constraints of LLSM, with the objective to study physiology in live cells at subcellular level. We validated barrier function of our brain microvessel-on-a-chip by measuring permeability of fluorescent dextran and a human monoclonal antibody. One potential application is to investigate mechanisms of transcytosis across the brain microvessel-like barrier of fluorescently-tagged biologics, viruses or nanoparticles.

## Introduction

The blood–brain barrier (BBB) is a unique and highly selective vascular interface that separates the peripheral blood circulation from the neural tissue in order to maintain a homeostatic microenvironment within the central nervous system (CNS) that allows the neuronal network to function properly (Abbott et al., 2010; Park et al., 2019).

The BBB is a complex vascular structure with specialized endothelial cells as its core element, surrounded by extracellular matrix (ECM) and supporting cells, such as astrocytes and pericytes (Greene and Campbell, 2016). Brain microvascular endothelial cells (BMVECs) that line the capillaries of the BBB are of crucial physiological importance since they tightly control the molecular and cellular flux between the blood and the brain, thereby regulating regional changes in nutrients and oxygen levels (Daneman, 2012), maintaining brain energy levels (Bordone et al., 2019) and mediating the local immune response in the CNS (Abbott et al., 2010). BMVECs differ from those found in peripheral vasculature as they have no fenestration and exhibit restricted paracellular passage for water and hydrophilic solutes due to the presence of a unique array of tight junctions and adherens junctions between adjacent endothelial cells (Greene and Campbell, 2016). Moreover, BMVECs have specialized transcellular transport mechanisms ensuring only wanted substances being actively delivered to the brain, and have shown to express a number of broad-spectrum efflux pumps on their luminal surface which severely limit the uptake of lipophilic molecules, including small molecule drugs, from the blood through the endothelium into the CNS. These characteristic anatomical and functional features of the BBB determine its crucial protective role for the CNS (Mahringer et al., 2011; Shawahna et al., 2011).

However, these highly selective barrier properties also extremely limit the therapeutic efficacy of drugs and hinder the treatment of neurological diseases such as Alzheimer’s disease, multiple sclerosis, Parkinson’s disease, HIV infection and brain tumors (Pardridge, 2006). Beyond therapeutics’ insufficient brain exposure, the BBB also plays a major role in the underlying pathophysiology of many of these CNS disorders which are usually associated with vascular hyperpermeability, transporter deficiencies, or an increase in leukocyte adhesion molecules, resulting in an abnormal, uncontrolled movement of cells and neurotoxins across the BBB blood vessel walls (Pardridge, 2006).

For studies of barrier function and dysfunction, *in vivo* models are of highest physiological relevance since the BBB is embedded in its natural microenvironment. These models are, however, limited in their throughput. Furthermore, animal models may not predict BBB penetrance and efficacy of drugs in humans due to interspecies differences in the molecular composition of the BBB microvessels (Uchida et al., 2011; Song et al., 2020). Deciphering the underlying molecular mechanisms and performing translatable real-time quantitative assessments of drug transport across brain microvessels, such as screenings for BBB-penetrant therapeutic antibodies, are therefore greatly limited in an *in vivo* setting. In contrast, *in vitro* brain microvessels and BBB models offer faster, yet simplified approaches for targeted drug screening as well as for fundamental research, and importantly can be humanized to overcome translatability issues.

Human BBB organoids provide a model that enables maintaining endothelial cells in close juxtaposition. A limitation of this system, however, is that they essentially lack flow since microvessel-like structures cannot be formed in organoids, rather endothelium-lined spheres are generated which can negatively impact cellular viability (Urich et al., 2013). Traditional two-dimensional (2D) *in vitro* models such as the Transwell system, in which endothelial cells are cultured on semi-permeable membranes, have extensively been used for cell-based high-throughput screening assays and for studying basic BBB characteristics such as barrier permeability and transepithelial/transendothelial electrical resistance (TEER) (Abbott et al., 1992; Biegel and Pachter, 1994; He et al., 2014). These simplified systems lack simulation of blood flow conditions and have proved to insufficiently recapitulate *in vivo* phenotypes including the expression of key junctional proteins (such as claudin-5) and transporters (such as Glut-1 and insulin receptor) (Campisi et al., 2018). To overcome some of these limitations, several 3D microfluidic and organ-on-a-chip BBB and brain microvessel models have been developed enabling co-culture and fluid flow (Prabhakarpandian et al., 2013; Herland et al., 2016; van Der Helm et al., 2016; Wevers et al., 2018; Oddo et al., 2019; Park et al., 2019). Nevertheless, a number of these models exhibit other limitations such as non-physiological rigid ECM substrates, failure to feature blood vessel-like geometry and the lack of controlled flow that resembles the hemodynamic forces which is known to be crucial for microvascular function (Herland et al., 2016). Hence, there is an essential need for *in vitro* models that better mimic the brain microvessel environment including unidirectional flow, physiological shear stress, absence of artificial membranes, and presence of the cylindrical geometry typical of capillaries to facilitate the complex cell-cell interactions and physical ECM mechanics known to be intrinsic to the *in vivo* BBB and brain microvessels. In order to be capable of providing molecular mechanistic insights, these models need to also be compatible with advanced imaging and live 3D tracking of labeled molecules.

Along these lines, we describe here our efforts to develop and use an *in vitro* human brain microvessel-on-a-chip consisting of a 3D microfluidic model with a hollow channel in which a continuous monolayer of cells can grow at the interphase between the lumen and the underlying ECM. For our studies, we chose the brain microvascular endothelial cell line TY10 which is a well-established BBB model system (Takeshita et al., 2014; Spampinato et al., 2015; Shimizu et al., 2017; Wevers et al., 2018; Shimizu et al., 2019). Moreover, our system may be amenable to any other barrier-forming cell type to mimic vascular beds of different tissues or epithelial barriers. Our system allows controlled unidirectional flow, within the lumen of the artificial microvessel, of media including substrates of interest, for instance drug candidate biologics. An important characteristic of our device is its open design that also allows direct access of reagents from the surrounding space to the underlying ECM. We demonstrate the utility of this open design of organ-on-a-chip model by showing it is amenable for quantitative 3D live fluorescence imaging using spinning disk confocal or lattice light sheet microscopy (LLSM) and for high resolution electron microscopy. This model is set out to provide insights into molecular mechanisms involved in the transcytosis of biologicals at extraordinary detail which will further support the development of antibody-shuttle technologies across the human BBB. Detailed imaging, for example, can be very useful to follow endo-lysosomal trafficking in real-time, informing on fate of antibodies and viruses when entering endothelial cells, thus informing on better designs of biologics and viral vectors that more efficiently penetrate or inhibitors for the transcytosis of pathological viruses.

## Materials and Methods

### Cell Culture

Human brain derived microvascular endothelial cells (TY10 cell line) were isolated from normal brain tissue from a patient with meningioma, and immortalized with retroviral vectors harboring a SV40 large T antigen gene that is engineered to drive proliferation at 33°C (Sano et al., 2010; Maeda et al., 2013; Sano et al., 2013). The TY10 cell line has been used to model the BBB in previous studies (Takeshita et al., 2014; Karassek et al., 2015; Spampinato et al., 2015; Shimizu et al., 2017; Takeshita et al., 2017; Wevers et al., 2018). Cells were cultured at 33°C, 5% CO_2_ in T75 flasks BioCoat (Corning, 354485, MA, United States). TY10 cells were used between passage 17–25 and cultured in ScienCell complete endothelial cell medium (ScienCell, 1001, CA, United States). Cell detachment was performed using Accutase^®^ (Corning, 25-058-CI, MA, United States) when cells were ∼80–90% confluent before being seeded into the microfluidic devices. Cells were routinely tested for mycoplasma contamination and found negative.

### TY10 Stably Expressing eGFP

A lentiviral vector expressing a plasma membrane targeted eGFP (memGFP) containing a chimera of the N-terminal 41 amino acids of human myristoylated alanine-rich C-kinase substrate (MARCKS) fused to eGFP was made by co-transfection of a plasmid harboring memeGFP and Virapower Packaging Mix (Thermo Fisher, K497500, MA, United States) into 293T cells. Culture media was harvested 72 h later, cellular debris pelleted by low-speed centrifugation, and further clarified by 0.45 μm filtration with Millipore steriflip vacuum filters (EMD, SLHV033RS, MA, United States). The supernatant from the viral preparation was added to a flask of TY10 cells during passaging (4 ml of viral supernatant preparation mixed with 4 ml of cell suspension were added to a T25 Corning BioCoat flask) and allowed to incubate for 24 h at 33°C before switching back to the normal feeding schedule of every other day. The cells were sorted by flow cytometry for eGFP positive cells after 10 days in culture and subsequently expanded and maintained as described above.

### hmAb

The recombinant monoclonal human IgG1 antibody (produced by Biogen) was expressed in CHO cells and purified through Protein-A Affinity Chromatography. The purified protein was fluorescently labeled with Alex Fluor^TM^ 568 and 647 protein-labeling kits (Thermo Fisher Scientific, 10238 and A30009, MA, United States) to produce hmAb-AF568 and hmAb-AF647 following the manufacturer’s protocol.

### Microvessel-on-a-Chip

#### Fabrication of Silicon Wafer

**FIGURE 1:**
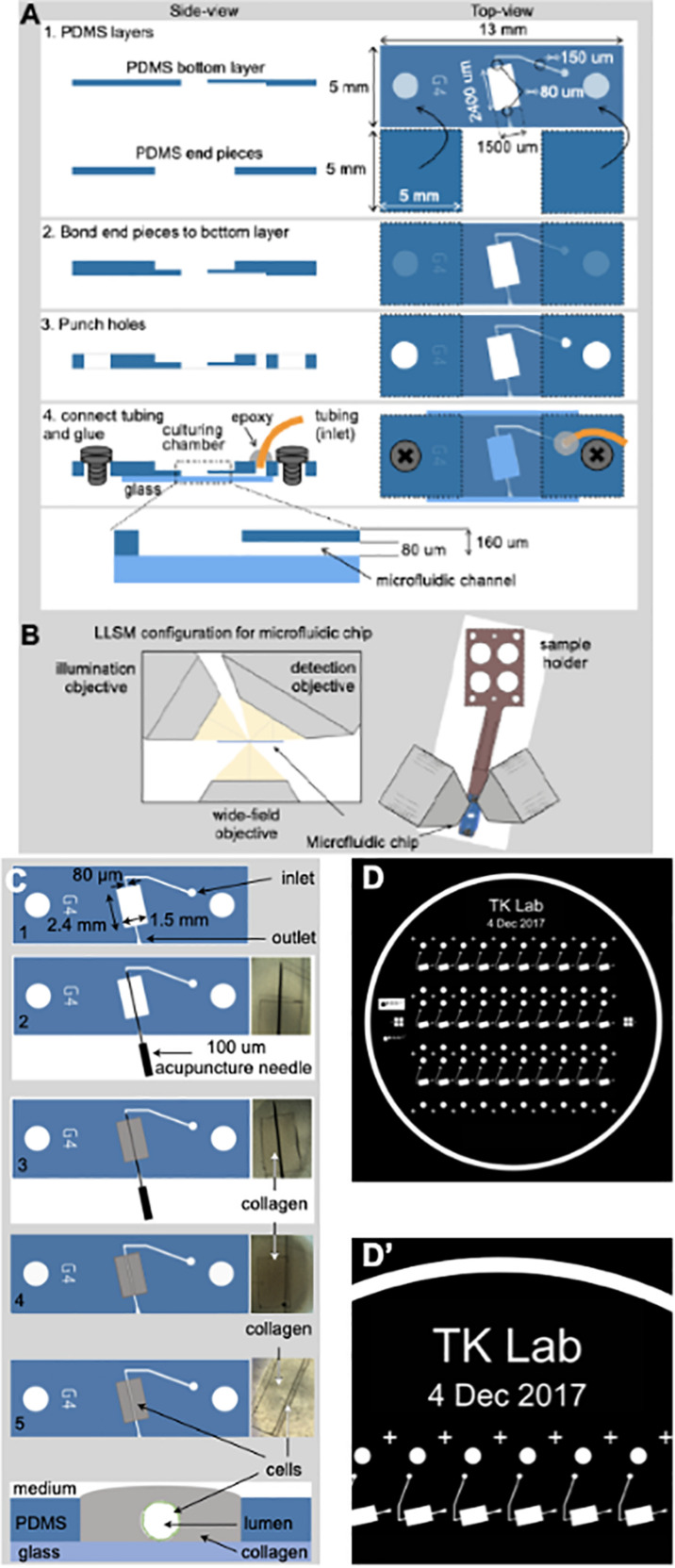
Fabrication of the microvessel-on-a-chip. **(A)** Schematic representation of the sequential steps used to fabricate the microvessel-on-a-chip. The open design permits direct access of aerated medium from the top to the collagen containing embedded cells while at the same time allowing flow of medium along the tubular channel with endothelial cells surrounding the boundary between the lumen of the artificial microvessel and the collagen. See associated Supplementary Movie 1 for guidance on the fabrication of the microvessel-on-a-chip. **(B)** Arrangement of the microvessel-on-a-chip within the available optical path between the illumination and detection objectives of the lattice light sheet microscope (LLSM). A sample holder is used to place the microvessel-on-a-chip under the LLSM objectives and to facilitate fine adjustments of its position. The chip can also be placed on a glass coverslip and placed in an inverted microscope for all modalities of conventional confocal microscope (not shown). **(C)** Steps used to create an artificial microvessel (1–5). An acupuncture needle of 100 μm diameter is placed between the inlet and outlet (2). After the collagen gelled (3), the needle was pulled (4), resulting in a void channel (5) in which endothelial or epithelial cells of choice, here brain endothelial cells, are seeded. The cross section on the bottom shows the final disposition of the brain endothelial cells (green) that line the inner surface of the collagen lumen, representing the artificial brain microvessel. **(D,D’)** Images of the mask template used to create the silicon wafer then used to prepare the PDMS mold.

Photomasks with the chip design depicted in Figure 1 were designed with AutoCAD (AutoDesk Corp., CA, United States) and printed by CAD/Art Services, Inc. (OR, United States). Molds of 80 μm depth were produced in a clean room by photolithography using standard protocols and SU-8 2050 photoresist (Microchem, now Kayaku Advanced Materials, Inc., MA, United States). The 3″ in diameter silicon wafer (University Wafer, 447, MA, United States) was cleaned by sequential solvent treatment in a Headway spin coater at 500 rpm. Each of the following solvents were squirted for 1 min onto the rotating wafer while moving a cotton swab across the surface: isopropyl alcohol, acetone, methanol, pure water. After dehydration on a hotplate at 150°C for 5 min the wafer was activated by oxygen plasma at 130 mTorr and 100 W for 5 min. A walnut sized amount of SU-8 2050 was placed on the wafer fixed in the Headway spin coater and an even film was generated by the following spin conditions: step 1: 500 rpm, 5 s, ramp 100 rpm/s; step 2: 2700 rpm, 30 s, ramp 300 rpm/s; step 3, 0 rpm, 10 s, ramp 300 rpm/s. After 30 min incubation at room temperature (RT), the soft bake was performed at 5 min 65°C and 20 min at 85°C following UV exposure in a MJB3 Mask Aligner (Süss-MicroTec, Munich, Germany) for 10 s at 25 mW/cm^2^. The post-exposure baking was the same as for soft baking. The mold was developed for 10 min in 20 ml MicroChem SU-8 developer, rinsed with isopropyl alcohol and blown dry prior silianization by incubating the wafer in a desiccator under reduced pressure in presence of an aluminum cup with 3 drops of (tridecafluoro-1,1,2,2-tetrahydrooctyl) trichlorosilane (Gelest, Morrisville, PA, United States). Hard bake was performed on a hotplate at 150°C for 15 min.

#### Production of the Microfluidic Device

Microfluidic devices were subsequently produced by soft lithography; Sylgard 184 elastomer Polydimethylsiloxane (PDMS) was mixed with curing agent (Sylgard 184 silicone elastomer kit, Dow Corning, Midlands, United States), at a 5:1 ratio in a mixer including a 2 min de-foaming step before pouring it onto the master silicon wafer designed by our lab and spin-coating at 400 rpm for 40 s. The utilized speed yielded a PDMS film of 160 μm thickness that was degassed in a vacuum desiccator for 10 min and cured in an oven at 65°C for 1 h. The PDMS film was peeled off the master and placed in a plastic petri dish at 65°C overnight to fully cure. To remove non-crosslinked oligomers within the PDMS that can leach out and contaminate the culture medium (Halldorsson et al., 2015), cut PDMS slabs containing the embossed microstructures and 5 × 5 mm end pieces were cleaned by contact with Scotch tape and subjected to organic solvent extraction (Lee et al., 2003). PDMS slabs were incubated in a sealed jar on a rocker for 24 h at RT in each of the following solvents and in this order: triethylamine, toluene, ethyl acetate, and acetone (all ACS grade and from Sigma-Aldrich, MO, United States). To evaporate the acetone, the PDMS slabs were placed on an accordion-shaped aluminum foil rack to reduce contact with any surface and incubation at 100°C for 2 h. Extracted PDMS remains hydrophilic for prolonged times after plasma-activation as it does not contain uncured oligomers (Lee et al., 2003; Kim and Herr, 2013). The PDMS extraction step significantly improves the bonding and microfluidic properties. The PDMS pieces of the chip were placed embossed featured down on a fluorinated ethylene propylene (FEP) sheet and exposed to air plasma at 700 mTorr, 30 W for 1.5 min using the PDC-001 plasma cleaner (Harrick Plasma) to bond the 5 × 5 mm end pieces before punching 1 mm holes for the tubing. PDMS pieces were rinsed with isopropyl alcohol and blow-dried prior plasma-bonding to the #1.5 glass coverslip that was cleaned by incubation in isopropyl alcohol, acetone and 0.5M KOH for 30 min each in a sonication water bath, rinsing five times in pure water and blown dry with filtered nitrogen gas.

To promote bonding, the chips were placed on a hot-plate at 150°C for 15 min. We tested several glues to fix the Tygon microbore tubing, 0.010” × 0.030” OD (Cole-Parmer, EW-06419-00, IL, United States) on the chip and SLOW-CURE^TM^ 30 min epoxy (Bob Smith Industries; BSI206, CA, United States) resulted in the sturdiest connection after overnight incubation and further allowed us to remove air bubbles in the epoxy after mixing through centrifugation for 30 s at 14,000 x g in a tabletop centrifuge. The chip was activated by air plasma treatment as above and further cleaned by injecting sequentially 0.5 ml of each acetonitrile, purified water, 0.5 M KOH and again water.

To functionalize both glass and PDMS surface with primary amine groups, the chips were silanized by pipetting 0.5 ml of a fresh 1% aqueous solution of 3-(Ethoxydimethylsilyl) propylamine (Sigma, 588857, MO, United States) on the chip and incubation for 15 min at RT before rinsing with twice with 1 ml pure water. Subsequently, the surfaces were further functionalized by adding 0.3 ml 2.5% glutaraldehyde (Electron Microscopy Services, 16200, PA, United States) and incubation for 15 min before the devices were rinsed extensively with pure water. The Schiff bases formed on proteins after glutaraldehyde immobilization are stable without further reduction, as has been demonstrated in surface-protein conjugation (Kim and Herr, 2013). To further facilitate adoption of the chip design, a detailed video with step-by-step instructions for chip fabrication has been added (see Supplementary Movie 1).

#### Formation of Lumen and Collagen Matrix

A Pluronic F-127 (Sigma, P2443, MO, United States) passivated 100 μm acupuncture needle was inserted from the outlet toward the inlet of the brain microvessel-on-a-chip to provide the required scaffold for the culturing matrix as indicated in Figure 1C. The selected size of the acupuncture needle should prevent the leakage of unpolymerized collagen into the microfluidic channel of a smaller diameter (80 μm). A hydrogel consisting of extracellular matrix (ECM) proteins made of a final concentration of 7.0 mg/ml Type I rat tail collagen (Corning, 354249, MA, United States) was used in all experiments. To make 200 μl of hydrogel solution, 39 μl of Endothelial Cell Medium (ECM basal media with no FBS; 1001b, ScienCell, Carlsbad, CA, United States), 1 μl of a basic solution (1.0 N NaOH, Sigma) and 14 μl of 10X Ham’s F-12 (Thermo Fisher, 31765092, MA, United States) were added to 135 μl of the collagen I.

We found that the collagen gel tended to delaminate from the PDMS culturing chamber as soon as we started the flow, and so we enhanced the standard collagen matrix protocol by adding Genipin^®^ (Sigma, G4796, MO, United States). Genipin is a crosslinking agent that covalently attaches to primary amino groups exposed on protein surfaces (Sung et al., 1998). Furthermore, Genipin monomers form covalent intermolecular crosslinks that in the case of a collagen matrix results in bridging adjacent fibers at points of contact (Yoo et al., 2011; Chan et al., 2014). Thus, to achieve a stiff and resilient collagen matrix we mixed collagen with Genipin prior pipetting it into the culturing chamber of the chip. The solution of 135 μl collagen, 39 μl ECM, 14 μl 10X Ham’s F-12, 1 μl 1N NaOH and 1 μl 20 mM Genipin was gently mixed and incubated on ice for a period of 5–10 min to get rid of any air bubbles which might generate during the mixing step, before being added to pre-chilled chips kept on ice for at least 15 min. The devices were subsequently incubated at 37°C to allow gel formation of the collagen matrix. Genipin improved the stability of the PDMS-collagen interaction such that delamination was never observed for up to 12 days. After removal of the acupuncture needle, non-reacted Genipin was quenched by covering the top of the collagen brain microvessel-on-a-chip with PBS containing 1 mM Tris pH 8.0 in PBS in addition to flowing the same solution through the cylindrical lumen for 15 min at 1 μl/min. Chips were then washed with 3 ml of PBS alone (added to the top of collagen) and flow of PBS alone for 15 min at 1 μl/min. Prior to cell seeding (see below), a solution containing complete ECM medium was injected to the lumen for 15 min at 1 μl/min.

#### Cell Seeding

**FIGURE 2:**
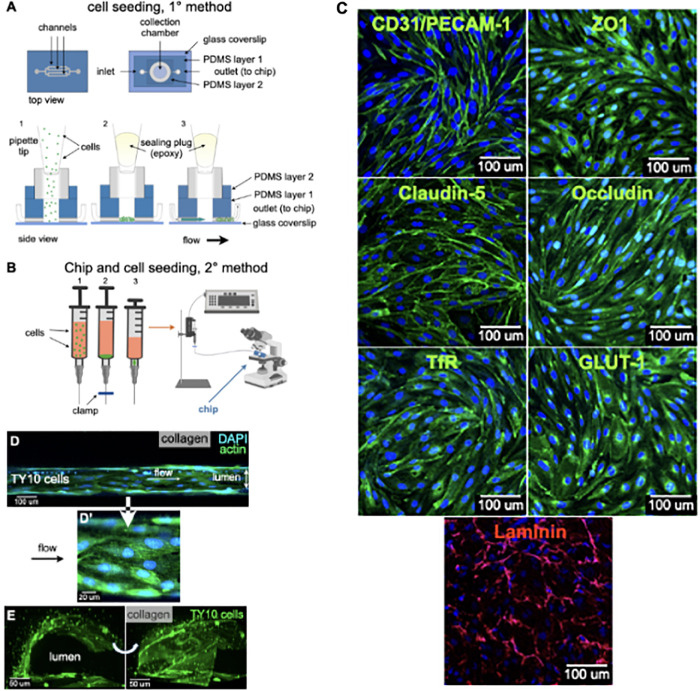
Cell seeding procedures and formation of a human brainmicrovessel using TY10 cells. **(A)** Cell seeding, 1st method. Representation of the steps used to increase the cell concentration before injection into the chip. Cells are allowed to settle by gravity (left and central panels) in the cell seeder and then injected as a bolus into the hollow lumen. Prior to injection, the chamber is capped with a plugged pipette tip (central panel). **(B)** Cell seeding, 2nd method. Representation of the steps used to inject cells into the chip in a more controlled and uniform manner compared to the 1st method. Cells are allowed to settle at the bottom of a syringe, and are then delivered into the hollow lumen at constant flow controlled by a syringe pump. **(C)** Immunostaining of TY10 monolayers: endothelial cell marker PECAM-1/CD31, transporters Glut-1 and transferrin receptor (TfR), and tight junction proteins ZO-1, Occludin and Claudin-5. Staining for laminin provides evidence that TY10 cells deposit extracellular matrix while in culture. Scale bar, 100 μm. **(D)** Representative image of a chemically fixed sample of TY10 cells after they were grown in the brain microvessel-on-a-chip with medium flowing from left to right at 1 μl/min for 7 days. Volumetric image was obtained using a spinning disk confocal microscope. Maximum z-projection is shown for a sample stained with DAPI (nuclei, blue) and phalloidin (actin, green). Scale bar, 100 μm. **(D’)** Representative image of a chemically fixed sample of TY10 cells after they were grown in the microfluidic-on-a-chip with medium flowing from left to right at 1 μl/min for 7 days. Single plane image was obtained using a Zeiss 710 confocal microscope. The image highlights the linear organization of actin stress fibers along the axis of flow. Sample stained with DAPI (nuclei, blue) and phalloidin (actin, green). Scale bar, 20 μm. **(E)** Representative volumetric image of a live sample of TY10-eGFP cells expressing membrane bound eGFP obtained using spinning disk confocal microscopy. The image highlights the organization of the cells as a monolayer at the boundary between the lumen of the artificial microvessel and the collagen scaffold. The cells on the bottom illustrate cells growing between the glass slide and the lumen. Panels are rotated 90-degrees from each other. Scale bar, 50 μm. See associated Supplementary Movie 2.

To line the lumen with TY10 cells and generate a perfused microvessel-like structure, two strategies were used to ensure uniform cell seeding. In the first strategy (cell concentrator chip, Figure 2A), we designed a gravity-based microfluidic cell concentrator to reach a sufficiently high density of cells for seeding of the collagen lumen using minimal cell concentration. A PDMS chip whose single channel splits up into four microchannels that merge again into a single channel after 5 mm was used as bottom layer with a central 2.5 mm collection chamber. To securely fit a 25 mm long silicon tubing of OD 4 mm/ID 2.5 mm, a second PDMS layer with 4 mm hole was bonded as a lid and the tubing was fixated with epoxy glue. The inlet of the concentrator chip was connected via tubing to a syringe pump and the outlet to the brain microvessel-on-a-chip. TY10 cells were resuspended to 0.1 million cells/ml and transferred into a 1 ml syringe. The cells settled by gravity within 15 min at the collection chamber on the bottom glass surface which was passivated with 0.01 mg/ml Poly-D-Lysine-PEG to prevent the cells from sticking to the glass. A plug comprised of an epoxy filled pipette tip was inserted into the central tubing to prevent upwards flow before initiating the flow. Applying flow through the microfluidic channel resulted in shear force that pushed the cell bolus into the tubing leading to the culturing chamber of the brain microvessel-on-a-chip.

In the second strategy a simplified procedure was implemented in order to enhance the experimental turnover as illustrated in Figure 2B and its results section. In brief, TY10 cells were harvested and resuspended to 1 million cells/ml. A total of 900 μl of the cell solution was then mixed with 100 μl solution of collagen IV (Sigma, C5533, MO, United States), fibronectin (Sigma, F2518), and laminin (EMD Millipore, AG56P) at 5:1:1 concentration ratio before transferred into a 1 ml syringe with BD Luer-Lok (BD, 309628, NJ, United States). The syringe was then hanged vertically to allow cell settling by gravity for 10–15 min with no flow. Cell seeding was initiated under flow for about 15–20 min at 1 μl/min. Cell seeding was monitored by visual inspection using a microscope to observe the brain microvessel-on-a-chip placed inside a petri dish kept under sterile conditions.

After either procedure, chips were incubated at 37°C for a minimum of 4 h before being perfused with fresh ECM media via positive pumping to wash out the unattached cells. The chips were maintained under continuous unidirectional flow at a rate of 1 μl/min in a cell culture incubator at 37°C, 5% CO_2_. Confluent TY10 monolayers were formed typically after 72 h and the brain microvessel-on-a-chip devices were used for subsequent analyses following 7 days after seeding incubation in all experiments.

### Actin Staining in the Microvessel-on-a-Chip

The collagen samples including the lumen were fixed and permeabilized using BD Cytofix/Cytoperm Kit (BD, 554714, CA, United States) according to the manufacturer’s protocol. In brief, Cytofix was added to a 1 ml syringe and perfused through the microvessel at a flow rate of 1 μl/min for 30 min before changing to the Cytoperm buffer (diluted 1:10 in PBS) for 1 h at 1 μl/min. The flow was then stopped and the brain microvessel-on-a-chip placed on a well of a six well plate in 2 ml of Cytoperm buffer (diluted 1:10 in PBS) overnight. The microvessel was stained with AlexaFluor488 Phalloidin (Thermo, A12379, at 1:100 dilution) and NucBlue (Thermo, R37606, 1 drop/500 μl) by perfusing the dyes in Cytoperm buffer at 1 μl/min through the microvessel for 1 h, followed by washing with 0.1% BSA in PBS buffer for 4 h at 1 μl/min. Images were collected using Zeiss LSM 710 microscope (10x objective) through the bottom glass slide.

### Immunofluorescence

TY10 cells were seeded in a 24-well glass bottom plate (In Vitro Scientific, P24-0-N, CA, United States), coated with rat tail collagen type I (50 μg/ml final concentration in 1% acetic acid) at 1 × 10^5^ cells per well. Cells were allowed to proliferate for 3 days at 33°C, 5% CO_2_ before they were switched to 37°C, 5% CO_2_ for 3 days to initiate differentiation of TY10 cells. Cells were fixed with 4% PFA (Thermo Fisher Scientific, 50-980-487, MA, United States) for 10 min at 4°C and then blocked with 10% normal goat serum serum (Thermo Fisher Scientific, 16210064, MA, United States) for 30 min at RT. Staining was performed under permeabilized conditions using BD Perm/Wash Buffer (BD Biosciences, 554723, CA, United States). Primary antibodies were incubated for 3 h at RT. The following primary antibodies were used: anti-CD31 (Thermo Fisher, MA3100, 1:100), anti-ZO1 (Thermo Fisher, 40-2300, 1:60), anti-Occludin (Thermo Fisher, 40-4700, 1:200), anti-Claudin 5 (Thermo Fisher, 35-2500, 1:50), anti-Glut-1 (Thermo Fisher, PA1-1063, 1:100), anti-TfR (Thermo Fisher, A-11130, 1:25) and anti-Laminin (abcam, ab11575, 1:100). The following secondary antibodies were incubated for 45 min at RT: goat anti-mouse AlexaFluor 488 (Thermo Fisher, A11001, 1:1000), goat anti-rabbit AlexaFluor 488 (Thermo Fisher, A11008, 1:1000), and goat anti-rabbit AlexaFluor 568 (Thermo Fisher, A11011, 1:1000), supplemented with Hoechst 33342 (Thermo Fisher, H3570, 1:2000). For imaging, Zeiss LSM 710 Confocal and Leica SP5 Confocal microscopes (40x objective) were used, and image processing was performed in ImageJ (NIH).

### Barrier Integrity Assay

#### Microvessel-on-a-Chip

Chips were washed with ECM culture medium (once with 1 ml added to the top of collagen followed by a flow at 1 μl/min for 15 min) to ensure proper flow profiles during the subsequent barrier integrity assay. Next, all medium was aspirated from the chip and 1 ml of medium without fluorescent compound using Gibco^®^ FluoroBrite^TM^ DMEM (Thermo Fisher, A1896701, MA, United States) was added. Medium containing 25 μg/ml of 10 kDa FITC-dextran (Sigma, FD10S, MO, United States) or hmAb-AF568 was added through the inlet at a flow rate of 1 μl/min for all the experiments. The inlet was connected to microvessels with and without TY10 cells and image acquisition was started. Leakage of the fluorescent substrate (dextran or hmAb) from the lumen of the microvessel into the adjacent collagen matrix was imaged using a spinning disk confocal microscope with 40x water immersion objective. The fluorescence intensity profiles and ratios between the fluorescent signal in the basal and apical region of the microvessel tube were analyzed using MATLAB (MathWorks, MA, United States). Apparent permeability (Papp) was used for quantifying diffusional permeability as described (Yuan et al., 2009). In brief, Papp was calculated by analyzing total fluorescence intensity in the imaged 2D area of the lumen and collagen and then applying Papp = (1/ΔI) (dI/dt)_0_ (r/2), where ΔI is the increase in total fluorescence intensity upon adding labeled dextran or labeled hmAb to the lumen, (dI/dt)_0_ is the temporal initial rate of linear increase in intensity as the labeled molecules diffuse out of the microvessel into the surrounding collagen matrix, and (r) is the radius of the microvessel (100 μm for our brain microvessel-on-a-chip). All experiments were carried out at *n* = 4–6; exact numbers are mentioned per experiment in figure captions. Graphs were plotted using GraphPad Prism 6 (GraphPad Software, CA, United States).

#### Transwell

Transwell inserts (Falcon, 08-771-8, MA, United States) were coated with 50 μg/ml rat tail collagen type I (Corning, Cat# 354236) in 1% acetic acid for 1 h and then washed with PBS prior to cell seeding. Per 12-well transwell insert, 125,000 TY10 cells were seeded in 1 ml ECM (ScienCell, 1001, CA, United States), and 2 ml ECM was added to the bottom chamber (Falcon, 08-771-22, MA, United States). Cells were allowed to proliferate at 33°C, 5% CO_2_ for 3 days, then switched to 37°C, 5% CO_2_ for another 3 days for differentiation, with media changes every other day. Transwell inserts without cells were used as control. On the day of the permeability assay, media was refreshed in both chambers and 1 mg/ml final concentration of 10 kDa FITC-Dextran (Millipore Sigma, FD10S, MA, United States) was added to the top chamber. The plate was incubated for 1 h at 37°C, 5% CO_2_ after which samples of 100 μl were taken from the bottom chamber and transferred to a black/clear bottom 96-well plate. Fluorescence intensity was determined with a plate reader set at excitation 490 nm and emission 535 nm.

### Spinning Disk Confocal Imaging

Imaging was done using a Marianas spinning disk confocal microscope (3i, Colorado, United States) with the water immersion objective lens LD C-Apochromat 40x/1.1 (Carl Zeiss, Jena, Germany). The images consisted of 512 x 512 pixels with a pixel size of ∼333 nm. The EMCCD camera settings (gain, speed, intensification, and exposure) and laser power were maintained throughout the imaging experiments. Images were acquired using SlideBook 6 (3i, Colorado, United States) and data analysis carried out using SlideBook 8 and custom-made software using MATLAB 2017A (Natick, MA, United States). For the analysis of heat maps and Papp, ROIs were the entire original field of view.

### LLSM Imaging

A microvessel-on-a-chip fabricated on 8^∗^5-mm rectangular #1.5 glass coverslip was picked with forceps, and placed in the sample bath of the 3D LLSM. The sample was imaged in a time series in 3D using a dithered multi-Bessel lattice light-sheet by stepping the sample stage at 200 nm intervals in the s-axis equivalent to ∼104 nm translation in the z-axis. Each 3D stack corresponded to a pre-deskewed volume of ∼80 μm x 120 μm x 47 μm (800 × 1200 × 451 pixels). The sample was excited with a 488-nm laser (∼100 mW operating power with an illumination of ∼77 μW at the back aperture), a 560 nm laser (∼100 mW operating power with an illumination of ∼176 μW at the back aperture) and a 642nm laser (∼100 mW operating power with an illumination of ∼121 μW at the back aperture) to acquire 451 imaging planes, each exposed for ∼44.1 ms and recorded with two Hamamatsu ORCA-flash 4.0-V2 cameras; thus, each 3D image took ∼60 s to acquire. The inner and outer numerical apertures (NAs) of excitation were 0.513 and 0.55, respectively. The overall 3D volume of ∼240 μm × 880 μm × 180 μm was obtained by stitching together 165 (3 × 11 × 5) 3D stacks, with an overlap of 40 and 9 μm in the *y*-axis and *z*-axis, respectively.

### Transmission Electron Microscopy

The collagen matrix including the lumen was washed 3 times with PBS and then fixed by immersion in 5 ml of fixing solution (2.5% glutaraldehyde, 2% sucrose, 50 mM KCl, 2.5 mM MgCl_2_, 2.5 mM CaCl_2_ in 50 mM Cacodylate buffer pH 7.4) (Sigma) and kept at 4°C, overnight in the dark. Fixed collagen samples were washed 3 times with a solution containing 50 mM PIPES pH 7.4 (Sigma, P6757, MO, United States) kept in ice and then they incubated for 2 h in ice and in the dark in a freshly prepared staining solution (SSI) made of 1% OsO4 (Electron Microscopy Sciences, 19190), 1.25% potassium hexacyanoferrate (II) (Sigma, 455989) and 100 mM PIPES pH 7.4. Samples were rinsed 3 times with ice-cold water and incubated again for a second time for 30 min in ice and in the dark with a freshly prepared staining solution II (SSII). SSII was prepared by 1:100 dilution of SSI in a freshly prepared 1% thiocarbohydrizide (Electron Microscopy Sciences, 21900, PA, United States). Finally, the samples were washed for 3 times with ice-cold H_2_O and then incubated overnight in the dark in 1% uranyl acetate (Electron Microscopy Sciences, 22,400, PA, United States) at 4°C.

For the dehydration and embedding step, the fixed and stained samples were first washed 3 times with ice cold water and then subjected to dehydration with a 20-50-70-90-100% ethanol – 100% acetone dehydration series. Samples were then infiltrated overnight, at 4°C with 50-50 acetone-Epon812 epoxy resin (Electron Microscopy Sciences, 14120, PA, United States). Next day, the samples were washed 3 times with 100% Epon812 and then kept in an oven for 36 h at 60°C. Sections of 60–70 nm in thickness were cut transversally to the long axis of the lumen and imaged with a JEOL JEM 1200 EX TEM microscope with a voltage of 80 KV and a nominal magnification of 15,000.

### Statistical Analysis

StatsDirect 3 (Liverpool, United Kingdom) was used for one-way ANOVA and student’s *t*-test analyses with Bonferroni *post-hoc* correction. Data were presented as mean ± standard error of the mean (SEM) where (^∗∗∗^) denotes a statistically significant difference with *p* < 0.001 and (ns) indicates a statistically non-significant difference.

## Results and Discussion

We engineered an open design microfluidic chip to generate a 3D microphysiological model of the human brain endothelial microvessel readily accessible to optical imaging. The unique characteristics of this novel brain microvessel-on-a-chip are the open design of the cell culture chamber and a cylindrical hollow lumen amenable to continuous unidirectional flow within a casted gel of extracellular matrix (ECM) components. The open system allows direct access of the collagen matrix for efficient exchange of gases and medium in addition to readily access to optical imaging while cells growing with continuous unidirectional flow at the interphase between the casted gel and the lumen mimic the environment of a microvessel.

### Fabrication of the Microvessel-on-a-Chip

Figure 1A graphically summarizes the sequential steps used to build a microvessel-on-a-chip model. It is based on sequential bonding using soft lithography (Bischel et al., 2013) of thin layers of optically clear PDMS on top on a glass microscope slide. The geometry and dimensions of the microvessel-on-a-chip were optimized for its use with three major complementary forms of live 3D optical imaging, spinning disk confocal, lattice light sheet microscopy (LLSM) and the recently developed variant, LLSM modified with adaptive optics (AO-LLSM) (Gao et al., 2019). We chose to include access to LLSM and AO-LLSM because these imaging modes have revolutionized fluorescence optical microscopy providing volumetric imaging with unprecedented high spatial and temporal precision with minimal bleaching and phototoxicity (Liu et al., 2018).

Spinning disk confocal microscopy is performed through the glass slide at the bottom of the microvessel-on-a-chip, while LLSM or AO-LLSM are carried out from the open top as illustrated in Figure 1B. The device is also suited for chemical fixation and the sample preparation required for high-resolution electron microscopy visualization.

**FIGURE 3:**
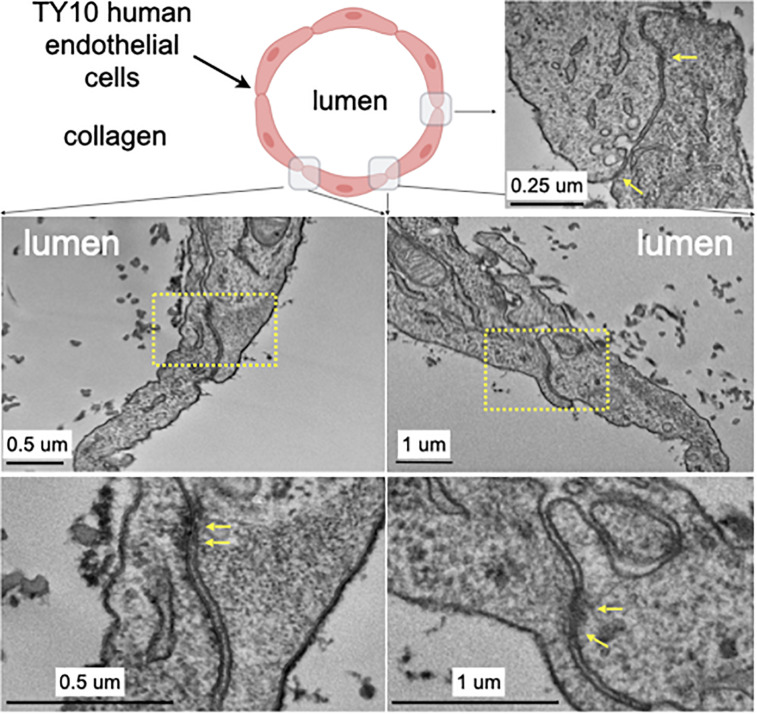
Transmission electron microscopy highlighting the appearance of junctions between TY10 endothelial cells within the brain microvessel-on-a-chip. The upper left panel shows a schematic cross section of the brain microvessel-on-a-chip highlighting the location of the monolayer of TY10 endothelial cells at the interface between the lumen of the microvessel and the collagen. The representative images in the bottom panels derive from junctions between opposite ends of adjacent cells. The yellow arrows highlight electron-density characteristic of tight junctions. Scale bars with corresponding magnifications are indicated.

**FIGURE 4:**
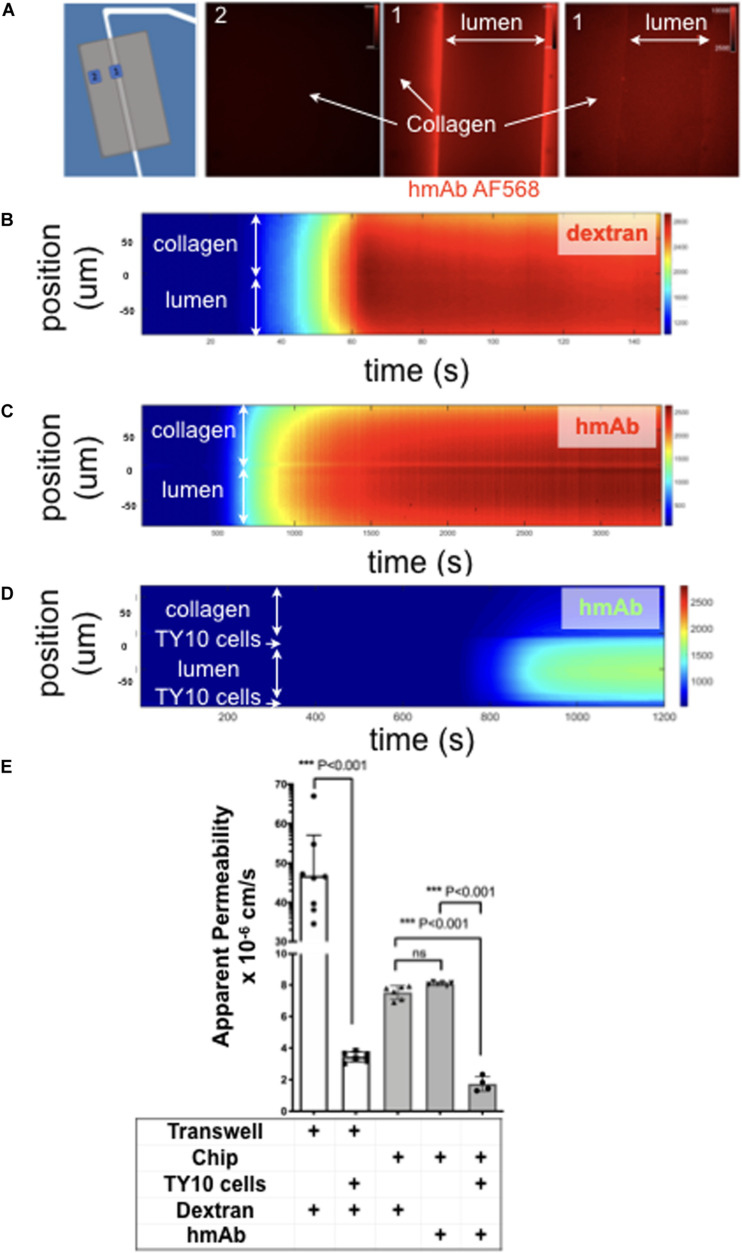
TY10 cells establish a functional barrier in the brain microvessel-on-a-chip. **(A)** The left panel is a schematic representation of the experimental setup used to determine the apparent permeability of fluorescent solutes diffusing between the lumen of the artificial microvessel and the collagen in the absence and presence of TY10 cells. The boxed numbered areas represent typical regions imaged using spinning disk confocal microscopy. The fluorescent images are of hmAb-AF568 (red) applied at constant flow (1 μl/min) for 20 min in the absence (left and central panels) and presence (right panel) of 1 mM TRIS in addition to Genipin (a chemical crosslinker) added to stabilize the collagen matrix (see Methods). Genipin followed by 1 mM TRIS treatment dramatically decreased the non-specific retention of the antibody by the collagen. **(B–D)** Heat map representation of the fluorescence intensity of 10 kDa FITC-Dextran **(B)** or antibody hmAb-AF568 **(C)** diffusing from the lumen through the collagen as a function of time obtained at a flow of 1 μl/min. The significant decrease in the amount of antibody that passes through the endothelial cell layer is highlighted in panel **(D)**, demonstrating that TY10 cells form a functional barrier in the brain microvessel-on-a-chip. See associated Supplementary Movies 3–5. **(E)** Apparent permeability data for 10 kDa FITC-Dextran or hmAb-AF588 obtained from experiments carried with a traditional 2D transwell (*n* = 8 without TY10 cells and *n* = 6 with cells) and with the brain microvessel-on-a-chip (see **B–D**) without (n = 6) and with (*n* = 4) cells. The experiments compare the apparent permeability of the soluble molecules in the absence or presence of cells between the two compartments of the transwell, or between the boundary of the lumen and collagen in the brain microvessel-on-a-chip. One-way ANOVA and student’s *t*-test analyses with Bonferroni *post-hoc* correction was used to identify significant differences between samples. Error bars indicate SEM.; ***p ≤ 0.001, ns: non-significant.

The consecutive stages used to generate the cylindrical hollow lumen within the casted gel followed by seeding of endothelial cells on the wall of the lumen are depicted in Figure 1C and described in detail in methods. It involved first placing an acupuncture needle between the microfluidic inlet and outlets (Figures 1C, 2) followed by casting a collagen matrix (Figures 1C, 3), gentle removal of the needle after collagen gelation (Figures 1C, 4) and ending with cell seeding under flow (Figures 1C, 4). A Pluronic F-127 passivated 100 μm acupuncture needle was inserted from the outlet toward the inlet to provide the required scaffold for the culturing matrix. Collagen type I (7 mg/ml) has been used to assemble the culturing scaffold. The chosen diameter of the needle enables the device to recreate artificial microvessels where an endothelial monolayer is formed against a collagen matrix and is stably maintained by shear stress and surface tension. See Supplementary Movie 1 for a brief video with step-by-step instructions to facilitate the construction by other laboratories of the microvessel-on-a-chip.

### Cell Seeding and Establishment of an Endothelial Microvessel

Cell seeding was performed with two methods. The first one involved use of a cell-concentrator chip designed and operated as indicated in Figure 2A. Cells in suspension were placed on a pipette tip linked to the top of the cell-concentrator, allowed to settle by gravity for 15 min to a cell density of ∼0.1 million/ml, and cells then injected into the hollow lumen with the aid of a syringe pump. Before activation of the syringe pump, we replaced the pipette feeding cells with an epoxy-plugged pipette as a way to prevent backflow. Afterward the bolus with cells reached the hollow lumen, flow was then stopped allowing cells to settle for 24 h so they could attach to the internal walls of the cylindrical lumen of the chip. TY10 cells were grown for 7 days under flow, at which point they established a monolayer and hence were ready for imaging experiments. We used TY10 cells to model the endothelium of the BBB, but any other endothelial or epithelial cell type may be also used to establish a barrier-forming cell model.

The second, and in our hands preferred cell seeding method (Figure 2B), involved use of a 1 ml syringe driven by a mechanical syringe pump. A solution containing ∼ 900 μl of 1 million/ml cells in medium mixed with 100 μl of a solution containing laminin, fibronectin and collagen type IV to promote cell attachment, was placed in a vertically oriented syringe and cells allowed to settle for ∼10–15 min. Afterward, flow of 1 μl/min was applied for 10–15 min in order to inject the cells into the lumen of the chip; cells were then allowed to settle and attach to the internal walls of the lumen for 4 h at 37°C. As with the first method, cells were then grown for 7 days at a flow rate of 1 μl/min, before their use for imaging. This simpler cell seeding method is particularly advantageous for cases in which the cellular supply might be limited such as when using primary cells or iPSC-derived cells from patients.

Extent of seeding was optically monitored with the aid of an inverted microscope by direct inspection of the device placed inside a closed petri dish to ensure sterility. Cellular viability was determined for both cell seeding methods using exclusion of Trypan Blue. These experiments indicate that the two methods have comparable results (viability of 91% and 96% for methods 1 and 2, respectively). In our hands, method 1 was associated with a 100% success rate (*n* = 8) while method 2 had a lower success of 87% (*n* = 29). Of note, success rate has been defined by a complete cell seeding of the lumen from the first round, resulting in full coverage of the lumen with endothelial cells, without the need for a second round of cell seeding.

To summarize, the two methods are associated with a comparable cell viability. Method 1 has a higher success rate and requires 10x less cells (0.1 million/ml vs. 1 million for method 2) but at the expense of longer incubation time (24 h vs. 4 h required for method 2) and extra time and cost associated with manufacturing of the cell concentrator microfluidic device. For these reasons, we have mainly opted for method 2 in this study, but intended to offer the reader a second option if cell supply is limiting, for instance.

### TY10 Cells as a Model for BBB Endothelium

In the brain, the basement membrane surrounding the endothelial cells of the brain vasculature is comprised of fibronectin, laminin (Aumailley et al., 2005) and collagen type IV (Hartmann et al., 2007). Indeed, *in vitro* monolayers of endothelial cells grown on a matrix containing fibronectin, laminin, and collagen type IV exhibit enhanced TEER, suggesting a role for these molecules in promoting the formation of tight junctions (Tilling et al., 1998, 2002; Gautam et al., 2016). To mimic the physiological BBB microenvironment and presumably also to enhance the seeding efficiency in the brain microvessel-on-a-chip, we injected before cell seeding a solution containing fibronectin and laminin for 30 min at 1 μl/min. TY10 cells were allowed to settle for 24 h at 37°C before starting the flow at 1 μl/min.

One of the major challenges of developing physiologically relevant *in vitro* brain microvessel models is the availability of suitable brain-derived cells of endothelial origin and of human origin in particular. Primary human brain endothelial cells or cells differentiated from induced pluripotent stem cells (iPSC) derived from control or diseased patients are preferred for *in vitro* brain microvessels and BBB models. Use of primary cells is restricted to very low passage numbers to prevent down-regulation of the unique features of the BBB (Reichel et al., 2003). More general, the difficulties in collecting and purifying these cells can considerably limit their use and reliability, as well as reproducibility (Bernas et al., 2010). Immortalized brain-derived cell lines can have great advantages such as accessibility and convenience of use especially for optimization purposes despite that some of the available lines might not exhibit all BBB characteristics (Kuhnline Sloan et al., 2012; Eigenmann et al., 2013; Wong et al., 2013). Nonetheless, certain cell lines may still exhibit the required properties for some pathophysiological and medicinal applications in a fit-for-purpose approach.

We therefore chose immortalized human brain derived TY10 microvascular endothelial cells that have been used to model the human BBB in a number of previous studies (Takeshita et al., 2014; Spampinato et al., 2015; Shimizu et al., 2017, 2019; Wevers et al., 2018). TY10 cells are immortalized and proliferate at 33°C, and stop growing and acquire a phenotype of primary brain endothelial cells at 37°C (Maeda et al., 2013). These cells harbor a spindle-shaped morphology, express markers typical for brain endothelial cells, and express P-glycoprotein (P-gp) irrespective of passage number (Sano et al., 2010, 2013). These cells also express the endothelial marker PECAM-1/CD31, transporters Glut-1 and transferrin receptor (TfR), and tight junctions claudin-5, occludin, and zonula occludens (ZO)-1 (Figure 2C). TY10 cells have well characterized barrier-forming features and correlation between tight junction expression and barrier function in TY10 cells is well-established in the literature (Spampinato et al., 2015; Shimizu et al., 2017, 2019; Takeshita et al., 2017). We also provide evidence that TY10 cells secret components of the extracellular matrix by staining for laminin (Figure 2C).

As depicted in the representative fluorescence microscopy image of a chemically fixed sample stained for actin and DNA shown in Figure 2D, TY10 cells grew as a monolayer at the interphase between the cylindrical lumen and the collagen matrix in the microvessel-on-a-chip. Cultured for 7 days with constant flow at 1 μl/min of media, they appeared elongated along the flow axis, in agreement with previous findings (Ohashi and Sato, 2005; Aird, 2007). We have also shown in Figure 2D an enlarged region showing elongated actin stress fibers along the axis of flow in the lumen of the microvessel. This confirms the overall elongated organization of the TY10 cells grown in the chip under conditions of unidirectional flow. Importantly, most TY10 cells failed to align when grown in the commercial Mimetas OrganoPlate^®^ platform that subjects the cells to bidirectional instead of unidirectional flow of medium (Figure 1D; Wevers et al., 2018). This is an important characteristic since shear stress is known to play a role in regulating signaling cascades (Conway and Schwartz, 2012), enhancing the expression of key genes associated with transporters and junctional proteins (Cucullo et al., 2011), and plays a pivotal role in BBB regulation (Neuwelt et al., 2008; Neuwelt et al., 2011). Further confirmation for the cell organization was obtained using TY10 cells stably expressing soluble eGFP grown in a similar way and then imaged by live cell fluorescence microscopy 3D imaging (Figure 2E and related Supplementary Movie 2).

We further characterized, at the ultrastructural level, the TY10 brain microvessel-on-a-chip established under unidirectional flow as a way to detect presence of tight junctions between adjacent TY10 cells grown at the lumen-collagen interphase by using transmission electron microscopy (TEM) (Figure 3). The representative images highlight the presence of a narrow gap between adjacent cells and the occurrence of tight junctions. Similar images were observed along most cell-cell contacts between TY10 cells imaged visualized in other regions from this and other TEM sections. The open design provides an ideal tool for advanced imaging strategies since sample preparation in closed microfluidic chips is a real challenge, as one needs to fix the cells *in situ*, and then remove the surrounding polymer and glass to gain access to the cells for further processing. In the study by Lemos et al. (2016), the TEM images presented in the paper were obtained by fixing the cells, freezing the tissue and then cutting the PDMS away while the chip was kept in a container of dry ice. The freeze-thaw cycle negatively impacts the microstructure of the cells as indicated by the loss of the continuity of cell membrane (Figure 6C; Lemos et al., 2016). When trying to extract the hydrogel directly without the freeze-thaw cycle, the lumen was often completely destroyed and no usable data were generated. The clear advantage of the open design chip is that the collagen and cells can be directly embedded for TEM imaging without the risk of damaging the cells with additional manipulations.

TEER is frequently used to evaluate the integrity of the tight junctions and barrier function of *in vitro* models of the BBB. Use of this approach is not practical for our brain microvessel-on-a-chip model because the geometric constrains prevent us from positioning electrodes on opposite sides of the endothelial monolayer between the cylindrical lumen and the collagen matrix. To circumvent this limitation, we capitalized on our ability to use fluorescence optical imaging with our device as a way to determine permeability coefficient across the endothelial monolayer and hence establish the extent of the barrier function of cells grown in the brain microvessel-on-a-chip. Using spinning disk confocal fluorescence microscopy, we determined the rate of transport of fluorescently tagged humanized monoclonal hmAb-AF568 (Figures 4A,C,D) or 10 kDa FITC-dextran (Figure 4B) across the lumen-matrix interphase in the absence or presence of cells. Unexpectedly, in the absence of cells, we found retention of hmAb-AF568 at the lumen-matrix interface (Figure 4A, central fluorescence image). This retention appeared to be due to capture of the antibody by unreacted Genipin (the stabilizing primary amine crosslinker used to stabilize the collagen matrix, see section “Materials and Methods”). Quenching the unreacted reagent (see section “Materials and Methods”) fully prevented the hmAb-AF568 capture (Figure 4A, right fluorescence image). As shown by the time-dependent heat maps depicted in Figures 4B,C, 10 kDa FITC-dextran and hmAb-AF568 freely diffused from the lumen toward the collagen matrix.

We designed our system with the goal of enabling the study antibody transcytosis, and thus the 10 kDa FITC-dextran was selected for permeability analysis since it has a similar hydrodynamic radius and appropriate equivalent size-exclusion range as antibodies, hence enabling us to perform a direct comparison.

The apparent permeabilities of hmAb-AF568 and 10 kDa FITC-dextran, determined as the flux through a given unit area under gradient concentration (cm s^–1^) were also similar (Figure 4E) as expected for molecules with comparable radius of gyration (1.86 and 5–6 nm, respectively) (Armstrong et al., 2004; Hawe et al., 2011). In contrast, presence of the TY10 monolayer at the lumen-matrix interphase significantly hindered the transport of hmAb-AF568 (Figure 4D), with a significantly lower apparent permeability (Figure 4E).

We include as a comparison data from a permeability assay with 10 kDa FITC-dextran carried out in a static transwell model. In these experiments, dextran was added to the top chamber above an endothelial TY10 monolayer, and was then sampled after 1 h from the bottom chamber. In a transwell insert without cells, dextran freely diffused from the top to the bottom chamber, while a significant reduction in dextran flux was observed in the presence of TY10 cells (13.5-fold, *p* < 0.0001), demonstrating that cells formed a functional barrier (Figure 4E). TY10 cells appeared tighter in the microfluidic device compared to the transwell model (average Papp values of 1.73^–6^ vs. 3.44^–6^, *p* = 0.004) (Figure 4E). It is noted that at baseline (no cell controls in transwell and microfluidic chip), the apparent permeability was lower in the collagen microvessel which is likely an effect from pore size, surface area or other parameters attributed to the semipermeable membrane of a transwell insert that do not allow a direct comparison between the two systems. We conclude that TY10 cells, when comparing to its respective no-cell control within each experimental system, established a significant barrier to antibody diffusion, in agreement with similar previous results obtained with these and other cells using different *in vitro* human brain endothelial cell models (Eigenmann et al., 2013; Wevers et al., 2018).

### Application of Advanced Lattice Light Sheet Imaging

**FIGURE 5:**
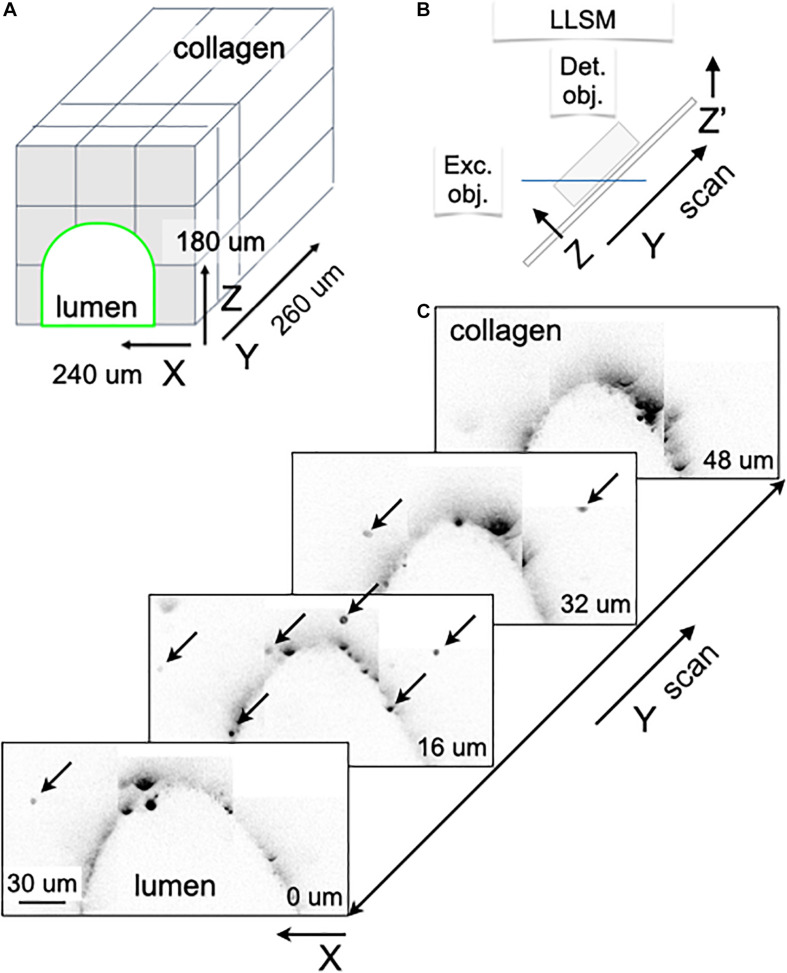
Visualizing IgG antibody diffusion in the microfluidic chip using LLSM. **(A)** Schematic representation of the volumetric imaging strategy used to visualize the lumen of the microvessel and surrounding volume within the chip using LLSM. The cubes represent the adjacent regions imaged by serial scanning of the sample using a distance of 100 nm between planes. **(B)** Schematic representation of the imaging set up used in LLSM. **(C)** Selected planes corresponding to the volumetric imaging obtained using LLSM of a sample containing 3 μm SPHERO^TM^ Goat anti-Human IgG coated polystyrene beads embedded in the collagen matrix. Before imaging, a solution containing hmAb-AF647 was perfused at 1 μl/min for 20 min. The fluorescent spots (marked by arrows in the selected planes located 16 μm apart) highlight beads with captured hmAb-AF647 located within the collagen matrix. The peak fluorescence intensity of the captured hmAb 2.6^+7^ ± 1.3^+7^ (mean ± SEM in arbitrary units) did not show a dependence with distance away from the lumen, consistent with efficient capture of the diffusing antibody. Scale bar, 30 μm.

Our microfluidic device is the only currently available design that can fit within the physical constraints of LLSM objectives (there is only 170 microns left to fit it the entire microfluidic device). In Figure 5, we provide a proof-of-concept that our design can fit and be successfully used to image the lumen and the surrounding collagen matrix not readily accessible with conventional microfluidic devices (Figures 5A,B).

We show our ability to image beads interspersed in the collagen matrix that have captured fluorescently tagged antibodies diffusing from the lumen of the artificial microvessel (Figure 5C). In this case, we placed 3 μm SPHERO^TM^ goat anti-human IgG coated polystyrene beads in the collagen matrix, to then capture hmAb-AF647 labeled antibodies perfused through the lumen of the microvessel within the chip; visualization was done using LLSM (Figure 5C, arrows). The peak fluorescence intensity of the captured hmAb 2.6^+7^ ± 1.3^+7^ (mean ± SEM in arbitrary units) did not show dependence with distance away from the lumen, consistent with efficient capture of the diffusing antibody.

In the future, we will capitalize on the recently developed lattice light sheet microscope modified with adaptive optics (AO-LLSM) that has significantly improved optical imaging capabilities to better visualize internal cellular compartments and investigate the transcytosis of antibodies and potentially other biotherapeutics across the endothelial barrier. This system has properties ideal for addressing other biological questions that can benefit from the advantages of LLSM and AO-LLSM because of their high temporal and spatial resolution combined with the very minimal bleaching even when the samples are imaged for prolonged times (Chen et al., 2014; Aguet et al., 2016; Liu et al., 2018).

Compared to traditional closed platforms, our open design facilitates harvesting of cells for gene expression profiling using RNAseq or quantitative PCR, protein expression by Western Blot or Mass Spectrometry, although the number of cells per microvessel may be limiting for some of these applications. Another advantage of the open chip design is the ease for chemical fixation and sample handling of the biological material located within the collagen associated with TEM, with high resolution volumetric imaging using Focused Ion Beam Scanning Electron Microscopy (FIB-SEM) and also with the newly developed modality of expansion microscopy combined with LLSM (Ex-LLSM) (Xu et al., 2017; Gao et al., 2019; Wassie et al., 2019).

Our results provide a supporting evidence showing how the open design of our microfluidic brain microvessel-on-a-chip device can be used to facilitate future studies of brain endothelial physiology at a subcellular level, particularly since cells can be grown under controlled unidirectional flow conditions. A next-generation model of the microvessel-on-a-chip could include addition of supporting cells such as astrocytes and pericytes to generate a more complex and physiologically relevant representation of the *in vivo* BBB. Further, our device is amenable to other barrier-forming cell systems found in vascular beds of peripheral tissues such as kidney or lung, for instance, or systems modeling like the gut epithelium.

The readily imaging accessibility of our open design is particularly suited for investigations of molecular transport mechanisms involved in the transcellular transport of biologicals, viruses or nanoparticles with the potential of providing insights at extraordinary level of detail. Although we exemplified here the implementation with the LLSM system, it is also designed to take advantage of AO-LLSM, which enables capture of high-resolution 3D movies of collective behavior of cells in a multicellular environment (Ji, 2017; Gao et al., 2019).

## Data Availability Statement

All datasets used and/or analyzed during the current study are available from the corresponding author TKi upon reasonable request.

## Author Contributions

MS, MD, GM, and TKi conceived the project. MS and GM were involved in all experiments. MD, MS, GM, SU, and TKi designed the brain microvessel-on-a-chip. RH helped with building the brain microvessel-on-a-chip devices under IK and MS supervision. GM and BO provided advice on all aspects of the cell biology associated with this project. BO performed immunostainings. TG performed dextran permeability assays in transwell. IK and MS performed spinning disk confocal microscopy. KO performed lattice light sheet microscopy under TKi’s supervision. GD helped with data analysis. GN performed electron microscopy. FS, YS, and TKa provided the parental TY10 cells and advised on cell culture procedures. TKi supervised the project. MS, BO, and TKi wrote the manuscript with comments from all authors.

## Conflict of Interest

GM and BO are employees and shareholders of Biogen. TG was employee and shareholder of Biogen at the time the study was performed. TKi is a visiting scientist at Biogen. The remaining authors declare that the research was conducted in the absence of any commercial or financial relationships that could be construed as a potential conflict of interest.

## Supplementary Material

MOVIE S1 Fabrication of the microvessel-on-a-chip. Simplified guide illustrating key steps during the fabrication of the microvessel-on-a-chip. The movie has been uploaded to FigShare^®^ and is available using this link: https://figshare.com/s/bb9ed44327f80a152abb.

MOVIE S2 Live cell imaging of a brain microvessel-on-a-chip. 3D rendition of a live sample of TY10-eGFP cells expressing membrane bound eGFP grown as a monolayer at the boundary between the lumen of the artificial microvessel and the collagen scaffold within the brain microvessel-on-a-chip. The volumetric image was obtained using spinning disk confocal microscopy.

MOVIE S3 Absence of a functional barrier between the lumen and the collagen matrix of the brain microvessel-on-a-chip. Time series of the fluorescence intensity presented as a heat map of 10 kDa FITC-Dextran diffusing from the lumen through the collagen as a function of time obtained at a flow of 1 μl/min. Data was obtained in the absence of a cell monolayer at the boundary between the lumen of the artificial microvessel and the collagen scaffold within the brain microvessel-on-a-chip.

MOVIE S4 TY10 cells establish a functional barrier in the brain microvessel-on-a-chip. Time series of the fluorescence intensity presented as a heat map of antibody hmAb-AF568 diffusing from the lumen through the collagen as a function of time obtained at a flow of 1 μl/min. Data was obtained in the presence of a monolayer of TY10 cells at the boundary between the lumen of the artificial microvessel and the collagen scaffold within the brain microvessel-on-a-chip.

MOVIE S5 TY10 cells establish a functional barrier in the brain microvessel-on-a-chip. Time series of the fluorescence intensity presented as a heat map of antibody hmAb-AF568 diffusing from the lumen through the collagen as a function of time obtained at a flow of 1 μl/min. Data was obtained in the presence of a monolayer of TY10 cells at the boundary between the lumen of the artificial microvessel and the collagen scaffold within the brain microvessel-on-a-chip.
